# Detection and prevalence of myocardial infarction early and late after heart transplantation detected by late gadolinium enhanced MRI

**DOI:** 10.1186/1532-429X-14-S1-P198

**Published:** 2012-02-01

**Authors:** Henning Steen, Eva Hofmann, Hugo A Katus

**Affiliations:** 1 Department of Cardiology, University of Heidelberg, Heidelberg, Germany

## Background

As recently shown, non-invasive late gadolinium contrast enhanced MRI (LGE-CMR) is able to detect myocardial infarction (MI) typical patterns in patients after heart transplant (HTX). Patho-physiologically, the underlying reason is an accelerated vascular immuno-atherosclerosis, the so-called transplant coronary artery disease (TCAD) which limits long-term survival of HTX pts. To date, there is only scarce data on the detection of early infarctions after HTX, the time course and the effects on myocardial function.

We hypothesized that LGE-CMR could detect TCAD-related MI in pts already early after the HTX procedure.

## Methods

123 patients (pts) were divided into group I (62 pts; HTX operation<2ys) and group II (61 pts; HTX operation>2ys). LGE-CMR (Gadolinium:0.2mmol/kg bw) was performed on a 1.5T Whole Body MRI scanner (Philips Medical Systems) and analysed blindly by two experienced observers. For anatomic LGE description, hearts were divided according to the 17-segment model. Areas of infarct-typical LGE patterns were defined as sub-endocardial LGE patterns of various transmurality and were quantified by delineation of hyper-enhanced areas related to the myocardial mass on LGE images (relative infarct size). Groups were compared using ANOVA. P-values ≤ 0.05 were considered statistically significant.

## Results

**Figure 1:**
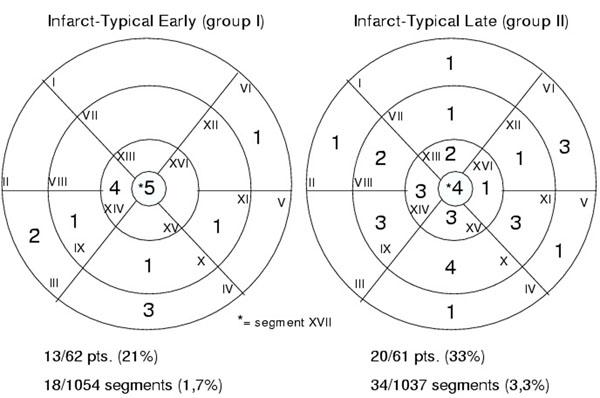
Localisation of early vs. late infarct-typical CE-MRI

In group I, already 21% of patients and 18 of 1054 segments (1.7%) showed infarct-typical LGE patterns. In contrast, significantly more patients (33%) of group II and 34 of 1037 (3.3% of segments, both p<0.01) segments were affected (figure 1). Mostly apical areas were affected and a decreasing tendency from apex to basal segments were observed. On a patient base, the number of affected infarct-typical segments increased significantly from 1.38 to 1.7, relative infarct size from 2.4% to 6.0%.

## Conclusions

LGE-CMR is a novel and sensitive imaging technique to detect infarct-typical MI in TCAD patients early and late after HTX. Unexpectedly, even in patients less than two years after the operation, there is already a noticeable prevalence of MI (21% of patients). These findings could have potential impact on a modified HTX patient risk stratification including LGE-CMR for tissue characterisation and detection of unknown MI.

## Funding

None.

